# Exploring Children’s Views on Important Park Features: A Qualitative Study Using Walk-Along Interviews

**DOI:** 10.3390/ijerph17134625

**Published:** 2020-06-27

**Authors:** Jenny Veitch, Elliott Flowers, Kylie Ball, Benedicte Deforche, Anna Timperio

**Affiliations:** 1 Institute for Physical Activity and Nutrition (IPAN), School of Exercise and Nutrition Sciences, Deakin University, Geelong 3220, Australia; kylie.ball@deakin.edu.au (K.B.); anna.timperio@deakin.edu.au (A.T.); 2 Institute for Health and Sport (iHeS), Victoria University, Melbourne 3011, Australia; elliott.flowers@vu.edu.au; 3 Department of Public Health and Primary Care, Faculty of Medicine and Health Sciences, Ghent University, C. Heymanslaan 10, 9000 Ghent, Belgium; Benedicte.Deforche@UGent.be; 4 Physical Activity, Nutrition and Health research unit, Department of Movement and Sport Sciences, Faculty of Physical Education and Physical Therapy, Vrije Universiteit Brussel, Pleinlaan 2, 1050 Brussels, Belgium

**Keywords:** parks, physical activity, social interaction, children, design

## Abstract

Parks are places where children can interact with others and engage in physical activity in a natural setting. Park visits can enhance children’s social, mental, and physical health. It is therefore important to better understand how parks can be designed to ensure optimal use by children. This qualitative study explored children’s perceptions of park features that may influence their park visits, park-based physical activity, and social interaction. Qualitative walk-along interviews were completed with 30 children (mean age 9.7 years (SD 1.3), female *n* = 16) in nine parks located in varying socioeconomic areas of Melbourne, Australia. As they walked through the park, children shared thoughts regarding characteristics that may influence their visitation, park-based physical activity, and social interactions. Features that would encourage visitation included: challenging and interesting play equipment; a pond and water play area; trees/greenery and shade; and full-sized basketball courts. Features most valued for physical activity included: sports courts, ovals, and equipment; open space; trees to climb; and nature/rocks. Features most valued for social interaction included: a large size; playgrounds; and picnic areas. Children offer unique and important views. Park designers should consider inclusion of these features, when (re)developing parks to support children to lead healthy and active lives.

## 1. Introduction

Parks are important locations for children to explore the natural environment, socialise with family and friends, engage in physical activity, and develop new skills such as fundamental motor skills [1], which are important for children’s physical, social, and mental well-being [2]. Engaging in physical activity outdoors has also been shown to have multiple social, psychological, and physiological health benefits [3]. Unfortunately, many parks are not being used to their full potential [4,5,6], and observational studies have shown that more than one-third of children are sedentary during visits to parks [4,5,6,7,8]. Based on accelerometry data, a study among 11–12-year-old children in the UK showed that the amount of time spent in moderate–vigorous-intensity physical activity in greenspace was low (2.4 min per weekday evening (3–10 p.m.) and 3.5 min on weekend days); however, the overall contribution to total outdoor physical activity was over 30% on weekdays and 46% on weekend days [8]. A New Zealand study using GPS showed that less than 2% of children’s weekly physical activity was located at urban parks [9]. Similarly, in Bristol, UK, only 2% of children’s outdoor activity was in green spaces such as parks and fields [10]. It is therefore important to better understand how parks can be designed to encourage greater use by children. According to social ecological models, health behaviours are influenced by individual, social, and physical environmental characteristics [11]. Physical environmental characteristics are likely to be particularly pertinent in terms of encouraging park visits and park-based physical activity and social interaction.

While access and proximity to parks and green space appear to be important determinants of park use among youth [12,13,14,15], empirical evidence also suggests that park quality [16], size and number of activity features [17], and specific facilities such as sports facilities, paths, trees, water elements, and maintenance [18] are important for encouraging visitation, physical activity, and social interaction among children. Not surprisingly, children are the greatest users of playground equipment, which promotes physical activity, motor skills, and social interaction [7,19]; however, even parks with play equipment remain underutilised by children [5]. Encouragingly, some studies that have observed children’s use of parks have shown significant increases in visits and active use following playground renovations [20,21,22], which suggests that park design has the potential to have a positive influence on visitation.

Children have different needs in regard to park features than adolescents, adults, and older adults [23]. Observational and activity tracking studies show that children engage in a variety of activities at parks such as playing and exploring [24], and these activities and park needs vary even throughout childhood. For example, a recent study exploring differences in features present in parks visited by children of different ages (3–5, 6–8, 9–11 years), found that older children (9–11 years) were more likely to visit larger parks that had more facilities and were further away from home compared with younger children [25]. It is therefore critical to ensure that evidence-based park design considers all demographic groups.

Social interaction is a key motivator for people to visit parks [26] and has a significant positive impact on social and mental health [27]. In an observational study among children in Australia, on average, children were observed playing or engaging with others (typically siblings, parents, or peers) in 85% of all observations [7]. The social environment, such as the presence of friends to play with at the park, has also been shown to influence the likelihood of children visiting parks as well as their engagement in park-based physical activity [28,29]. However, to our knowledge, no studies have examined specific park features or design elements that may influence children’s social interaction within parks. Furthermore, obtaining input from children directly about features that may encourage or discourage their park visits and active and social park use is critical for informing future park design [26].

Qualitative research can provide in-depth insights; however, the more traditional methods of individual or focus group interviews rely on children recalling past experiences. Conducting interviews while walking through the park (“walk-along interviews”) creates an interplay between the environment, the researcher, and the participant, which can help prompt in-depth discussion and facilitate contextual discussions about the park environment without relying on children to recall their previous park experiences [30]. This methodology has previously been used to capture children’s (aged 7–9 years) perceptions of their neighbourhood [31] and adolescents’ perceptions of the park environment [32].

A deeper understanding of the needs of children and features that they believe will encourage park visitation, physical activity, and social interaction within parks is essential to help park designers create parks that encourage and enhance park visits and support active and social play for children [33]. The aim of this qualitative study was to gain in-depth insights from children about park features that influence their park visitation, park-based physical activity, and social interaction using walk-along interviews in parks. 

## 2. Materials and Methods 

This research was part of a larger study (ProjectPARK) that examined park characteristics influencing park visitation, park-based physical activity, and social interaction among children, adolescents, and older adults. Following COREQ (Consolidated criteria for reporting qualitative research) guidelines [34], this qualitative study reports results from walk-along interviews completed with children aged 8–12 years between September 2017 and February 2018. 

A researcher interviewed children whilst they both walked through one of nine urban public parks located in metropolitan Melbourne, Australia. The parks were located in areas of varying socioeconomic status (SES; three parks in low SES, three parks in mid-SES, and three parks in high SES areas), as defined by the Australian Bureau of Statistics Socioeconomic Index for Areas [35].

To ensure that a range of potentially positive and negative park characteristics were observed during the interviews, the parks included in this study represented diversity in terms of size (1–30 ha), amenity, and condition. For example, across the nine parks the following characteristics were present: shady trees (*n* = 9); paths (*n* = 9); picnic tables (*n* = 9); barbecues (*n* = 8); playgrounds (*n* = 8); flying foxes (*n* = 7); toilets (*n* = 7); built shade (*n* = 7); basketball courts (*n* = 6); landscaping (*n* = 6); climbing structures (*n* = 5); sports ovals (*n* = 4); adventure playgrounds (*n* = 4); skate ramps (*n* = 3); cricket nets (*n* = 3); ropes course (*n* = 3); ponds (*n* = 2); water play area (*n* = 2); fitness equipment (*n* = 1); and table tennis tables (*n* = 1). All parks were open to the public at all times during the course of the study; none were fenced.

### 2.1. Participants

Children aged 8–12 years were recruited via their parents using multiple methods (number recruited presented in parentheses for each) including: flyers handed out at shopping strips located close to each of the selected parks (*n* = 3); flyers posted at local venues such as cafes (*n* = 3); advertisements targeting parents of 8–12 year olds on Facebook (*n* = 3); emails distributed to staff at the University (*n* = 2); and in-park recruitment (*n* = 19). Parents of children recruited by means other than in-park recruitment, were sent study details and once they had agreed to participate, they and their child met the research team at one of the selected parks on a specified day and time. For in-park recruitment, research staff approached parents who were accompanying children who appeared to be 8–12 years. They explained the study and invited participation if their child met the age range criteria and was English speaking. Where possible, research staff attempted to recruit an even mix of boys and girls. Overall, 14 parents who were approached in the park declined participation. Recruitment continued until no new information was obtained. All parents and children (*n* = 30) provided written consent. Ethics approval was obtained from the Deakin University Human Ethics committee (HEAG-H 94_2017). 

### 2.2. Procedure and Instrument

Firstly, the parent/guardian of each participant was asked to complete a brief survey to describe their child’s demographics, physical activity levels, and usual park visitation behaviour (Table 1). Parents reported their child’s age, sex, country of birth, dog ownership, school year, and duration at current address. For overall physical activity levels, parents reported how many days their child was physically active for at least 60 min per day in the past seven days and during a usual week [36]. This item has shown good agreement with accelerometers in predicting compliance to physical activity recommendations among youth aged 15–17 years [37]. They also reported the following with reference to their child’s park visits in the past three months: usual frequency and duration of park visits; usual activity levels during parks visits; usual accompaniment to the park: usual activities engaged in at the park (e.g., played ball games, played on playground, etc.) and mode of transport used to get to the most visited park. Parents also reported the frequency of their child’s usual social activity during park visits (i.e., met or talked to new people and known people; and participation in social events).

Secondly, one of eight research staff (four male, four female) interviewed the child whilst walking through the park together. The research staff completed training in qualitative data collection prior to conducting the interviews; they did not have existing relationships with any of the participants or report any biases or conflicts of interest. The interviews lasted between 7 and 20 min and were conducted in English. Voice recorders were worn by participants during the interview. At the start of the interview, children who had visited the park previously were asked why they visited that park, whereas those who had never visited the park were asked to describe things they may like to do during a visit to the park. All children were then asked to speak about what they liked and disliked within the park; what would make them want to be active or social here (i.e., “hang out” with other people); how they would change the park to make it better, and to make them want to be active or social; how being in this park made them feel; why they visit other parks (if they do); and to describe their ideal or perfect park. The researchers explained that there were no right or wrong answers and the children were the “experts”.

### 2.3. Data Analysis

Data from the survey were used to calculate descriptive statistics in Stata Statistical Software: Release 15 (StataCorp LLC, College Station, TX, USA). Qualitative data from the walk-along interviews were transcribed verbatim and analysed using NVivo 12 (QSR International Pty Ltd., Melbourne, Australia). Data analysis was guided by a summative content analysis approach [38]. A preliminary coding framework was established based on the interview questions, and this was adapted throughout the analysis and coding process. The walk-along interviews were read carefully, responses were then coded into frequently recurring subcategories and groups. The assignment of the subcategories and grouping was performed by two researchers (J.V. and a research assistant), and disagreements were discussed until agreement was reached.

## 3. Results

Overall, 30 interviews (2–5 interviews per park) were completed across the nine parks (13 in parks in low SES areas, 6 in parks in mid-SES areas, and 11 in parks in high SES areas). The average age of participants was 9.7 years (SD 1.3), 53% were female, 40% owned a dog, and 73% had previously visited the park where the interview was conducted (Table 1). A high proportion of children were regular park visitors, with 77% of parents reporting their child had visited a park at least once per week in the past three months and 50% of children usually spent between 30 and 60 min in the park. Most children (87%) usually visited the park with a parent or adult family member, and half visited with a sibling or friends. More than half of the parents (60%) reported that their child usually walked to the park they visited most often, and 53% usually engaged in mostly moderate-intensity activities (e.g., climbing a tree, riding a bike, playing at the playground) when at the park. Parents reported that the three most common activities their child performed when at the park were playing on the playground (70%), going for a jog/run (67%), and riding their bike (50%).

### 3.1. Walk-Along Interviews

Children stated that the main activities they either currently performed, or would be most likely to perform if they visited the park where the interview was conducted, were as follows: play on the playground; ride scooters; use the skate/BMX park; use the flying fox; be with friends; play soccer/cricket; walk around/relax; play tiggy (tag); play basketball; play in water play area; climb; or do school sport.

Children reported that the most common reasons for visiting “other” parks were the attraction of particular playground equipment (i.e., larger/more adventurous, greater variety); to use the skate park; to ride their bike; because the park was located close to home; they liked the trees/nature; and/or to have a barbecue. When one child, who was not a regular visitor to parks (i.e., parents reported their child visited parks about 2–3 times/month), was asked if they visit other parks they replied “Not really because there’s not many like really, really good parks near my area” (Boy, 8 years, park in high SES area).

*Well, they do have fences around their playgrounds, which feels a little bit safer and they have other equipment in the playground that these playgrounds don’t*.

(Girl, 10 years, park in mid SES area)

*I like that there’s sometimes a place with tonnes of trees that you can hide in and play in.I find that really fun*.

(Girl, 11 years, park in mid-SES area)

The words most commonly used when children described how they felt in the park were all positive and included happy, relaxed, excited, active, free, sporty, joyous, and comfortable.

*It makes me feel quite, almost active and adventurous*.

(Girl, 11 years, park in mid SES area)

*Well it makes me feel happy and it makes me feel like I want to go here more often. It makes me want to feel like to take all my friends here to play with them*.

(Girl, 9 years, park in low SES area)

*Relaxing, it’s good to get out of the house, get some fresh air and exercise. Yeah it’s nice*.

(Boy, 12 years, park in high SES area)

#### 3.1.1. What They Liked about the Park

When asked what they liked about the park, the most popular responses were playgrounds; trees/nature; climbing equipment; size; flying fox; basketball/netball courts; maintenance/aesthetics; walking/bike paths; grassy open spaces; skate parks; and sports ovals. In reference to the playground, they particularly liked the more challenging and “risky” equipment and having a variety of equipment.

*Probably the more risky things, the more risk taking things, not like full on dangerous, but they feel a little bit scary. A thrill*.

(Girl, 10 years, park in mid-SES area)

*I like the swings. They go quite high….. and well everybody in my class likes them, because they race from over there to here*.

(Girl, 9 years, park in mid-SES area)

*There’s like a lot of variety. Like it has monkey bars, the flying fox, slides*.

(Girl, 11 years, park in low SES area)

Nature was very important to many children.

*I like that it’s got lots of nature and greenery, and it’s really accessible to everyone. A lot of people can have fun here. Yeah. I love nature a lot. Nature is sort of like my number one thing in a park*.

(Girl, 10 years, park in mid-SES area)

*I like the hill. With my friends I like rolling down and getting up straight away really dizzy and trying to run off*.

(Boy, 11 years, park in high SES area)

*Yeah, the pond is very nice. I like it because it’s a little different, because if there was no pond in here, you could only see grass and trees. But then there’s a different element once you add the pond*.

(Girl, 10 years, park in high SES area)

*I like trees, water, stones and flowers because it’s natural and it’s a nice place to be in, and not like all roads and concrete*.

(Girl, 12 years, park in low SES area)

Having a larger sized park, bike paths, and sport ovals were also features children liked.

*I like it because it’s big. So that it’s not like a tiny park, like a tiny bit, and there’s not much space or things to do there*.

(Girl, 9 years, park in low SES area)

*I like there’s riding space and we can ride our bikes, scooters and roller blades*.

(Girl, 8 years, park in high SES area)

*I like the ovals because you can play football and soccer and there’s just things to do there*.

(Girl, 8 years, park in high SES area)

#### 3.1.2. What They Disliked about the Park

When speaking about things they disliked, the main comments related to the playground equipment. They commented that the equipment was too small and not challenging or interesting enough and sometimes not well maintained. They also spoke about a general lack of amenities such as taps, toilets, and rubbish bins, too much empty space with not enough equipment or planting and overall poor maintenance in regards to the landscaping and presence of rubbish.

*Well I think I’d make the zip climb and the flying foxes a bit longer. Maybe the swings higher up from the ground because they’re a bit low*.

(Girl, 9 years, park in low SES area)

*Like all the plants are really small and I like big plants that you can like play in and hide in and stuff*.

(Girl, 11 years, park in mid-SES area)

To encourage them to visit more often, children reported that they would add a water feature/pond with ducks and a water play area, improve maintenance (cleaner toilets, remove rubbish, improved maintenance of playground), provide more trees nature/greenery and shade, have a skate bowl and full-sized basketball courts, and more water taps and bins.

#### 3.1.3. Features That Encouraged Physical Activity

The main features that encouraged children to be active included: basketball/netball courts; open space and sports ovals; trees to climb and trees for hide and seek; nature/greenery/rocks; and playgrounds that are large and have things to run around on. Some children also spoke about being active when they visited the park with their dog.

*What would make you want to be active in this park?*

Respondent: *I only run around when I bring my dog. If I didn’t bring my dog, I would rather play tiggy with my friends and run around. Actually, I would like some more trees because when I play tiggy I get to go around the trees and stuff and play hide and seek*.

(Girl, 8 years, park in low SES area)

*So, what it is about this park that makes you want to be active?*

Respondent: *Maybe the forest area around here. I like running through it and the hill and the big open space to play soccer and run around on*.

(Boy, 11 years, park in high SES area)

To encourage them to be more active, the children suggested having more sports courts, equipment (i.e., soccer goals) and ovals, bike/walking tracks, and large playgrounds.

*Maybe add a few more like basketball courts… so if one person is already playing in one of them you don’t have to wait, and you can just go to go to the next one really easily*.

(Girl, 10 years, park in low SES area)

*How would you suggest changing this park to make you want to be more active here*?

Respondent: *Maybe a few more paths around*.

Interviewer: *OK, so if there were more paths, what would you do on the paths?*

Respondent: *You could do running or jogging, stuff like that*.

(Girl, 11 years, park in mid-SES area)

#### 3.1.4. Features That Encouraged Social Interaction

Playgrounds; picnic and barbecue areas with sufficient shelter, tables, and chairs; facilities suitable for a range of ages; large size, basketball/netball courts; grassy open space areas; trees/nature; and sports equipment (i.e., soccer goals, balls) were discussed as features that encouraged social interaction.

*What would make you want to meet up with your friends or family and be social?*

Respondent: *Well because it’s perfect scenery. Also, there’s lots of shade and lots of sun, and there’s lots of grassland where you can put your blanket for picnics and put it down and just eat. Or you could just get one of the benches*.

(Girl, 10 years, park in high SES area)

*Do you think your friends or family would want to come to this park with you?*

Respondent: *Yes, because like it’s a big space, and like if it’s my birthday I’ll invite a lot of people here, because it’s like fun for all ages*.

(Girl, 10 years, park in low SES area)

*Are there any ways you could change this park that would make you more likely to play with other friends you didn’t bring or make new friends?*.

(Boy, 11 years, park in high SES area)

Respondent: *Soccer balls because everyone could play*

*My school has this thing called a yarn circle. So, it’s basically [seats in a circle] where you sit around, and you can basically chat, or you can hop on the logs around the circle. It’s really fun, because you can just hang out with your friends, and it’s a place to relax or have fun*.

(Girl, 10 years, park in mid-SES area)

When one child, who was not a regular visitor to parks (i.e., parents reported their child visited parks about once/month), was asked if they thought their friends would come to this park they replied, “*Not really because they’re not really interested in being active*” (Boy, 8 years, park in low SES area). 

### 3.2. Description of the “Perfect Park”

When describing their “perfect” park, the following features emerged as being most important: playgrounds; green space/nature; large size; sports ovals, courts, and equipment; paths; water features; and things to climb. Overall, the most important feature of children’s “perfect park” mentioned by most children was a large adventure playground. Apart from it being high and large, children specified particular play equipment they wanted including long slides, large swings of all types including tyre and 360° swings, flying foxes, and monkey bars.

*A really big playground, like a big wooden structure with a high bit. It has lots of areas, and it’s like a maze, but it’s lifted off the ground. And there’s one part where it’s up really high and the playground has lots of pathways and stuff*.

(Boy, 11 years, park in mid-SES area)

*I guess it would be sort of like this but maybe I’d make stuff bigger and probably higher off the ground because I love to climb*.

(Girl, 9 years, park in low SES area)

Nature emerged as a top priority in children’s “perfect” park. This included trees to climb and play hide and seek, flowers, water, rocks, and nature trails.

*Maybe there could be like a little place where it’s like a kind of like a trail that you can go and that shows you like around the park. Well they could have a little wildlife area, that you could see lots of cool plants and things. Because we have something like that at my school. Well it’s just that it’s unique and other parks don’t have it, that I know of. I think people like me would really enjoy it and seeing all the wildlife*.

(Girl, 8 years, park in high SES area)

*Well it would probably have a pond, lots of trees, paths, and chairs obviously, flowers, and just like a lot of pathed areas. Not too much concrete, just stuff that makes it seem like a natural place*.

(Girl, 11 years, park in high SES area)

*Yeah, and maybe even a kitchen garden, like volunteers could come and organise a club where they plant food. They come once or twice a week and they can just grow stuff and bring it home and everything*.

(Girl, 10 years, park in mid-SES area)

Grassy open spaces also emerged as being an important component of their “perfect park”.

*Well I really like more space for grass because I get to run around and play fetch with my dog*.

(Girl, 8 years, park in low SES area)

*I think it should have quite a bit [green space] because that makes me feel really active and that I want to run all over it and stuff*.

(Girl, 11 years, park in mid SES area)

Both girls and boys repeatedly spoke about having challenging equipment to climb such as ropes, climbing frames, and towers, as well as the provision of multiple full-sized courts such as basketball courts and sports ovals.

*It would definitely have like a big rope climbing area. I’d have quite a few climbing frames. Lots of climbing stuff. Yeah just more things to climb on. I’d put lots of big rocks in to climb around on*.

(Girl, 8 years, park in high SES area)

*And how big would you want the climbing ropes to be?*

Respondent: *As high as possible. I’d love it like as high as they can go, to challenge myself*.

(Boy, 11 years, park in low SES area)

*Well maybe most of the things to climb on. Lots of monkey bars because I really like monkey bars and I’m good at doing lots of tricks*.

(Girl, 9 years, park in low SES area)

*It absolutely must have steep [skate] ramps, a basketball court, and a scooter shop. It would be close to home and it would have a massive basketball court*.

(Boy, 10 years, park in low SES area)

*Then all of this space would be saved for like I’d have a full-sized soccer pitch there, full-sized footy oval there, full-sized everything*.

(Boy, 11 years, park in low SES area)

Although mentioned less often, children also spoke about wanting a variety of features that suited people of all ages.

*Space for everyone to play with, like stuff. So, adults can have gym stuff, kids can have playground, and teenagers can have a skate park. And if they wanted to be sporty, like tennis courts, basketball courts, and golf*.

(Girl, 12 years, park in low SES area)

*A lot of stuff that family can do together. Not just a playground for kids, maybe one that a family could join into, like an adventure park*.

(Girl, 10 years, park in mid-SES area)

## 4. Discussion

Increasing children’s physical activity levels is a priority for public health [39]. Concurrently, increasing engagement in screen-based behaviours among children is reducing social interaction among youth, which has a negative impact on their social and mental health [40,41]. Improving parks and providing amenities that encourage children to visit parks, interact with others in these spaces, and engage in physical activity outdoors is potentially a long-term and sustainable way to improve children’s social, mental, and physical health [3]. This study provided an in-depth exploration of features that might promote park visitation, park-based physical activity, and social interaction from the child’s perspective. 

Children in this study typically visited parks at least once per week and were usually accompanied by parents, siblings, and friends. A key finding of this study was that the size, scale, and adventurousness of the playground are important for promoting visits, physical activity, and enjoyment. Additionally, results showed that children place value on nature as a location for play, particularly with friends, and the presence of friends was critical for encouraging park visitation. 

Overall, children had mostly positive things to say about spending time in the parks where the interviews were conducted. Even when children were asked about things they disliked about the park, they tended to report the scale of the playground equipment (e.g., wanting higher slides and swings, longer flying fox, etc.), rather than the absence of amenities or concerns regarding safety and cleanliness. These themes are distinct from those observed among other age groups, who more often report negative feelings and concerns towards the features and condition of parks and public open spaces [42,43]. 

Children repeatedly expressed the importance of playgrounds for encouraging visits, physical activity, and overall enjoyment. Survey responses from parents indicated that 70% of the children usually played on the playground during their park visits. Additionally, playgrounds were the most discussed feature throughout the interviews by a considerable margin and were particularly prevalent when children were asked to describe features they liked and their “perfect” park. This is consistent with previous research that has shown increases in park use and physical activity among children after playground upgrades [22,44]. Many responses from children indicated that they seek adventurous playgrounds or apparatus that contained an element of risk such as large swings, slides, flying foxes, etc.: “they feel a little bit scary. A thrill”. This is consistent with previous research among children [29,45]. 

Nature is also important for children, however unlike other populations, children see it as an opportunity for play, more so than for relaxation and social interaction. For example, children within this study referred to rolling down hills, hiding behind trees, and climbing on rocks. Children in the study also liked large open spaces so they could ride bikes, have fun, and play sports. This reinforces previous research that showed that the installation of a nature-based playground significantly increased visitation and park-based physical activity among children [20].

Consistent with previous research that has found the presence of family and friends, unplanned encounters with others, and social functions to support park usage and park-based physical activity [26,32], the social environment emerged as an important determinant of children being active. In previous studies, children have frequently spoken about how they went to the park or played in the street with friends and they would be active together [46]. Here children had clear ideas of features that they felt would encourage social interaction within parks, including large spaces, seating, or picnic areas. Children also demonstrated inclusivity in their emphasis on the importance of facilities that were not only personally appealing to them but also suitable to those of other age groups such as adults or older youth. This is an important consideration as children usually visit parks with friends, siblings, or adult family members [47].

Strengths of this study included the exploration of children’s views, which are often lacking yet provide invaluable insights [26], and the walk-along methodology, which created an interplay between the environment (both social and physical), the researcher, and the participant. Children 8–12 years old participated in this study. Including participants from this age band helped to generate a diverse set of themes relevant to a range of children including both older children and pre-adolescents. Conducting interviews while walking within the park means that participants can experience and interpret the park environment in the moment rather than recalling past park experiences which may be difficult, especially for children. In addition, the interviews were conducted in nine parks of varying size and amenity, a large percentage (43%) of interviews were conducted in parks located in low SES areas, and there was an even gender split among participants. These were further strengths that provided scope for a range of views and experiences to be explored. Limitations of this study include the high proportion of children who were regular park visitors. Overall, 77% of parents reported that their child had visited parks at least once per week in the past three months, and 63% of participants were recruited from within the park. Although seven participants were irregular park visitors, future research should consider exploring in-depth the views of irregular and non-park visitors to ensure parks are designed to meet the needs of all demographic groups. In addition, all parks were located in urban areas of Melbourne, so future research would benefit from examining perceptions of children living in rural areas. Finally, this study focussed on “in-park” features, so future studies may wish to examine children’s perceptions on factors impacting access to parks.

## 5. Conclusions

This research comprises an early step in better understanding park features that influence visitation, social interaction, and physical activity from the child’s perspective. Overall, the findings suggest that children are drawn to parks that facilitate play, have elements of risk/adventure, and are large enough to allow for a variety of physical and social activities. Children viewed nature favourably, particularly when it allowed for exploration and active play. Neighbourhood parks can facilitate a variety of social and physical activities for children. However, when (re)developing parks, designers need to consider exactly what features are important to encourage children to visit and be active and social in the park and should involve children in the planning and design process.

## Figures and Tables

**Table 1:** Demographic characteristics of participants.

	*N* = 30
**Age**, mean (SD)	9.7 (1.3)
**Sex, Female** (%)	53.3
**Country of birth, Australia** (%)	93.3
**Dog owner** (%)	40.0
**School year (%)**	
2	10.0
3	26.7
4	16.7
5	6.7
6	20.0
7	6.7
Missing	6.7
**Years at current address**, mean (SD)	9.9 (8.7)
**Number of days of physical activity (>60 min) in past week** (%)	
2	3.3
3	10.0
4	26.7
5	20.0
6	10.0
7	26.7
**Number of days of physical activity (>60 min) in a usual week** (%)	
1	3.3
2	3.3
3	10.0
4	26.7
5	16.7
6	23.3
7	16.7
**Usual frequency of park visits in past 3 months** (%)	
About once per week	26.7
2–3 times per week	50.0
About 2–3 times per month	16.7
About once per month	6.7
**Usual duration of park visits in past 3 months** (%)	
<30 min	10.0
30–60 min	50.0
>60 min	40.0
**Usual activity levels during park visits in past 3 months** (%)	
Mostly light activities	23.3
Mostly moderate activities	53.3
Mostly vigorous activities	6.7
Missing	16.7
**Usual accompaniment to the park** (%) ^a^	
Parent or other adult	86.7
Sibling(s)	50.0
Friend(s)	50.0
Dog	30.0
**Usual activities during park visits in past 3 months** (%) ^a^	
Went for a walk (excluding dog walking)	23.3
Walked the dog(s)	23.3
Went for a jog/run	66.7
Rode a bike	50.0
Played ball games	40.0
Did other exercise	6.7
Played on playground	70.0
Relaxed	13.3
Had a picnic/BBQ	30.0
Hung out with family	36.7
Hung out with friend(s)	43.3
Attended major event/celebration/birthday	16.7
Visited café/restaurant	16.7
Spent time in nature	23.3
**Mode of transport to most visited park in the past 3 months** (%)	
Walked	60.0
Jogged	6.7
Cycled	36.7
Public Transport	13.3
Car	40.0
Scooter	10.0
**Met or talked to new people during park visits in past 3 months** (%)	
Never	36.7
Once	20.0
2–3 times	20.0
4 or more times	23.3
**Met or talked to known people during park visits in past 3 months** (%)	
Never	16.7
Once	16.7
2–3 times	50.0
4 or more times	16.7
**Participated in a social event during park visits in past 3 months** (%)	
Never	36.7
Once	20.0
2 or more times	43.3

^a^ Multiple responses possible.
